# Monitoring multiple myeloma in the peripheral blood based on cell-free DNA and circulating plasma cells

**DOI:** 10.1007/s00277-022-04771-5

**Published:** 2022-02-01

**Authors:** Elisabeth K. M. Mack, Sören Hartmann, Petra Ross, Ellen Wollmer, Christoph Mann, Andreas Neubauer, Cornelia Brendel, Jörg Hoffmann

**Affiliations:** Department of Hematology, Oncology and Immunology, Philipps-University Marburg and University Hospital Gießen and Marburg, 35032 Baldingerstraße, Marburg Germany

**Keywords:** Multiple myeloma, Minimal residual disease, cfDNA, Next generation sequencing, Circulating plasma cells, Multiparameter flow cytometry

## Abstract

With the advent of novel, highly effective therapies for multiple myeloma (MM), classical serologic monitoring appears insufficient for response assessment and prediction of relapse. Moreover, serologic studies in MM are hampered by interference of therapeutic antibodies. The detection of malignant plasma cell clones by next generation sequencing (NGS) or multiparameter flow cytometry (MFC) circumvents these difficulties and can be performed in the peripheral blood (pB) by targeting circulating cell-free DNA (cfDNA) or circulating plasma cells (CPCs), thus also avoiding an invasive sampling procedure. Here, we applied NGS of VJ light chain (LC) rearrangements in cfDNA and MFC of magnetically-enriched CD138-positive CPCs (me-MFC) to investigate disease burden in unselected MM patients. Sequencing was successful for 114/130 (87.7%) cfDNA samples and me-MFC results were analyzable for 196/205 (95.6%) samples. MM clones were detectable in 38.9% of samples taken at initial diagnosis or relapse (ID/RD), but only in 11.8% of samples taken during complete remission (CR). Circulating MM plasma cells were present in 83.3% of ID/RD samples and 9.9% of CR samples. Residual disease assessment by NGS or me-MFC in samples taken during very good partial remission or CR was 80% concordant. Notably, 4/4 (NGS) and 5/8 (me-MFC) positive CR samples were from patients with oligo- or non-secretory myeloma. The time to progression was shorter if there was evidence of residual myeloma in the pB. Together, our findings indicate that our two novel analytical approaches accurately indicate the course of MM and may be particularly valuable for monitoring patients with serologically non-trackable disease.

**Supplementary Information:**

The online version contains supplementary material available at 10.1007/s00277-022-04771-5.

## Introduction

The treatment landscape for multiple myeloma (MM) has expanded substantially in the past few decades. Most importantly, proteasome inhibitors, immunomodulatory agents, and therapeutic antibodies have led to improved survival rates and highly effective combination therapies including these substances have been implemented as first-line therapies [1–3]. Moreover, nuclear export inhibitors and chimeric antigen receptor T cells have shown promising results in clinical trials for patients with relapsed or refractory disease [3, 4] and thus have been approved recently for MM. Therapeutic antibodies against CD38 and SLAMF7 such as daratumumab and elotuzumab are also increasingly applied as maintenance therapy due to their little side effects [5]. Yet, these antibodies interfere with well-established laboratory tests for monitoring MM, specifically protein electrophoresis and immunofixation to detect the disease-associated paraprotein, thus potentially leading to false positive results [6]. Hence, monitoring minimal residual disease (MRD) below the sensitivity threshold of serologic assays has gained in importance over the past years. Recent studies suggest that MRD monitoring by multiparameter flow cytometry (MFC) or next-generation sequencing (NGS) achieves sensitivity levels of 10^−6^ and can be applied to the vast majority of MM patients. There is evidence that MRD positivity predicts unfavorable outcomes even among patients achieving a complete response (CR) [7, 8].

Most previous MM MRD studies have investigated disease burden using bone marrow (BM) aspirates or biopsies [7], which requires an invasive sampling procedure. Liquid biopsies on the other hand involve analyses of circulating cell-free DNA (cfDNA) or circulating tumor cells (CTCs) and can be obtained by simple peripheral blood (pB) draws [9]. cfDNA plasma concentrations in MM patients are significantly increased not only in comparison with healthy individuals [10], but also in comparison to patients with solid tumors [11]. These findings underscore that cfDNA may enable noninvasive monitoring of MM. On the other hand, circulating myeloma plasma cells (MM-PC) represent a very rare target [12–14], so that it has been proposed to increase the sensitivity of cell-based assays by a preenrichment step using immunomagnetic or microfluidics techniques [15–17]. Liquid biopsies of MM patients have been investigated previously in pilot studies by different groups and these reports indicate that the quantification of immunoglobulin (Ig) heavy (IGH) and light chains (LC) in cfDNA as well as CTCs can mirror the course of the disease and response to therapy. [12, 13, 15, 18–22]. Moreover, parallel analyses of different types of MM liquid biopsies using NGS techniques have been reported before [23, 24]. However, we are not aware of any studies [25] that directly compared a molecular (NGS) and a cell-based (MFC) approach to detect MM in the pB to each other and to standard serologic monitoring. In this work, we present two novel technical modifications of pB-based methods for the detection of MM. Specifically, we investigated whether (1) NGS of Ig LC rearrangements only in cfDNA and (2) magnetic enrichment of CD138-positive (CD138^+^) cells before MFC measurement (me-MFC) enables reliable quantification of disease burden in both serologically detectable and undetectable MM.

## Materials and methods

### Patients and samples

This study investigated pB samples (cfDNA and/or whole blood samples) from 80 unselected MM patients, all treated at the Department of Hematology, Oncology and Immunology, Philipps-University Marburg, Germany. Patients were included irrespective of state of disease and previous therapies. Samples were collected between December 2015 and March 2018 (cfDNA for NGS) or, respectively, December 2015 and February 2020 (pB for me-MFC). There was no fixed schedule for sample collection. Disease/response state was assessed according to the IMWG guidelines [26] and patients were classified into the following response groups: stable disease (SD), progressive disease (PD), partial remission (PR), very good partial remission (VGPR), and complete response (CR). For the purpose of this study, stringent complete response was not separated from complete response and minor responses were classified as SD because of the low number of samples in this group (*n* = 2 in the cfDNA set and *n* = 3 in the me-MFC set). In addition to pB samples, 38 genomic DNA samples from BM biopsies, BM aspirates, or pleural effusions were subjected to NGS in order to facilitate the identification of MM-specific clones. For the comparison of immunophenotypes of circulating plasma cells (CPCs) and BM plasma cells, 17 BM aspirates and the corresponding pB of the same patients were analyzed in parallel by MFC.

### Immunomagnetic selection of CD138^+^ cells and multiparameter flow cytometry

Thirty milliliters of pB (K3 EDTA tubes, Sarstedt, Nümbrecht, Germany) were taken from each patient for immunomagnetic selection of CD138^+^ CPCs and MFC. All samples for me-MFC were processed freshly after a maximum storage of 48 h at room temperature. Leukocytes were isolated by dextran separation as follows: blood was suspended 5:1 in a 5% dextran (Sigma-Aldrich®, Munich, Germany) solution and allowed to sediment for 40 min at room temperature before leukocytes were removed from the upper phase and washed with phosphate-buffered saline (PBS, Invitrogen Darmstadt, Germany). The remaining erythrocytes were lysed 5 min at room temperature in an ammonium chloride containing lysing solution (10 × solution: 1.5 M NH_4_Cl; 100 mM NaHCO_3_; 10 mM disodium EDTA, distilled H_2_O). Labeling of total leukocytes with ferromagnetic monoclonal CD138 antibodies was performed according to the manufacturer’s instructions (Miltenyi Biotec, Bergisch-Gladbach, Germany). In brief, leukocytes were resuspended in 80 µl running buffer (Miltenyi Biotec) with 20 μl CD138 MicroBeads (Miltenyi Biotec) for 15 min at 4 °C protected from light. Afterwards, cells were washed with running buffer and separated with the autoMACS® Pro Separator (Miltenyi Biotec) in mode “posseld2” (repeated magnetic separation). Immunomagnetically enriched CD138 cells were resuspended in 100 µl PBS and transferred to a 5-mL polystyrene FACS-tube with fluorescence-antibodies in a dried-down layer (DuraClone-Technology, Beckman Coulter, Krefeld, Germany). The following antibody combination was used: CD81 Pacific blue (clone JS64), CD45 Krome orange (clone J33), CD38 FITC (clone T16), CD138 PE (clone B-A38), CD27 ECD (clone 1A4CD27); CD19 PC5.5 (clone J3-119), CD117 PC7 (clone 104D2D1), CD56 APC-AF700 (clone N901 (NKH-1)), CD200 APC-AF750 (clone OX-104) (all from Beckman Coulter). The cell suspension was incubated for 15 min at room temperature. After antibody staining, cells were washed with 3 mL PBS and centrifuged at 300* g* for 5 min. The cell pellet was resuspended in 500 μL PBS and measured on a Navios Flow Cytometer (Beckman Coulter). In total, up to 1 × 10^5^ cells were acquired. More than ten conclusive clustering CD38 and/or CD27 positive and CD138 positive events were classified as a significant number of circulating plasma cells. If a sample contained less than ten of such events, the result of the me-MFC analysis was categorized as “No PC.” If a sample contained two distinctive subsets of normal PC and malignant/aberrant PC with more than ten clustering events each, the result of the me-MFC analysis was categorized as “MM- and N-PC.” Aberrant plasma cells (myeloma cells) were proposed in case of at least two of the following seven immunophenotypically defined aberrancies: low to negative expression of CD45, CD19, CD81, and CD27 or aberrant positive expression of CD117, CD56, and CD200.

### DNA extraction

PB samples for NGS were drawn into Streck Cell-free DNA BCT® (Streck, Omaha, NE) blood collection tubes. The plasma was separated by centrifugation and cfDNA was isolated from plasma using the QIAamp Circulating Nucleic Acid Kit (QIAGEN, Hilden, Germany). cfDNA was eluted in 40 µl AVE buffer. Genomic DNA from bone marrow and pleural effusion mononuclear cells was extracted using the QIAamp DNA Mini Kit (QIAGEN, Hilden, Germany). DNA isolation from FFPE samples was carried out using the blackPREP FFPE DNA Kit (Analytik Jena, Jena, Germany). Concentrations of cfDNA as well as genomic/FFPE DNA samples were measured using the Qubit 3.0 fluorometer with the Qubit high sensitivity DNA assay (Invitrogen). Plasma concentrations of cfDNA were calculated assuming a plasma volume of 5 ml.

### Target amplification, library preparation, and sequencing

BIOMED-2 primer sets [27] were applied for amplification of Ig LC regions (IGK and IGL). Primers were purchased from Eurofins Genomics (Ebersberg, Germany). All primers targeting IGK or IGL, respectively, were mixed in a single tube and used for PCR in a final concentration of 10 nM, together with 0.4 µl AmpliTaq Gold (Applied Biosystems, Foster City, CA). A total of 10–20 ng cfDNA or 30 ng gDNA was used as an input. Amplification conditions were essentially as described [27]. The PCR product was cleaned up using AMPure XP beads (Beckman Coulter). Libraries were prepared using 30 ng of PCR products and the NEBNext® Ultra™ II DNA Library Prep Kit for Illumina® (New England Biolabs, Frankfurt am Main, Germany). Sample-specific barcordes (NEBNext® Multiplex Oligos for Illumina®) were added according to the manufacturer’s instructions. Libraries were quantified by qPCR with the NEBNext® Library Quant Kit for Illumina®. The quality and size distribution of sequencing libraries was analyzed on an Agilent 2200 TapeStation instrument (Agilent Technologies, Böblingen, Germany). Libraries were diluted to 4 nM and pooled in equal volumes. The library pool was diluted to 18 pM and combined with 1% of a 10 pM PhiX library (Illumina, San Diego, CA). Sequencing was performed on an Illumina MiSeq sequencing platform with 2 × 250 bp paired-end reads.

### Bioinformatics and statistics

Alignment of sequencing reads, clonotype assembly, and export of clonotypes was performed using MiXCR [28] (v2.1.10; downloaded from: https://github.com/milaboratory/mixcr/releases?after=v3.0.3). IGK and IGL repertoires were analyzed by VDJtools [29] (https://github.com/mikessh/vdjtools/releases/tag/1.1.8). Clonotype diversity was estimated using the bias-corrected Chao1 index (Chao1-bc) [30, 31], which has been applied previously for BCR- and TCR-repertoire analysis [32] and is already implemented in VDJtools. R version 3.6.1 (https://www.r-project.org/) and GraphPad Prism versions 7.03 or 8.11 (GraphPad Software, San Diego, CA) were used for statistical analysis. For statistical comparisons, samples taken at initial diagnosis (ID) and relapse (RD) and, respectively, SD and PD were grouped together. Agreement of NGS and me-MFC results was analyzed using GraphPad QuickCalcs (https://www.graphpad.com/quickcalcs/kappa1.cfm).

## Results

### Patient and sample characteristics

**Fig. 1:**
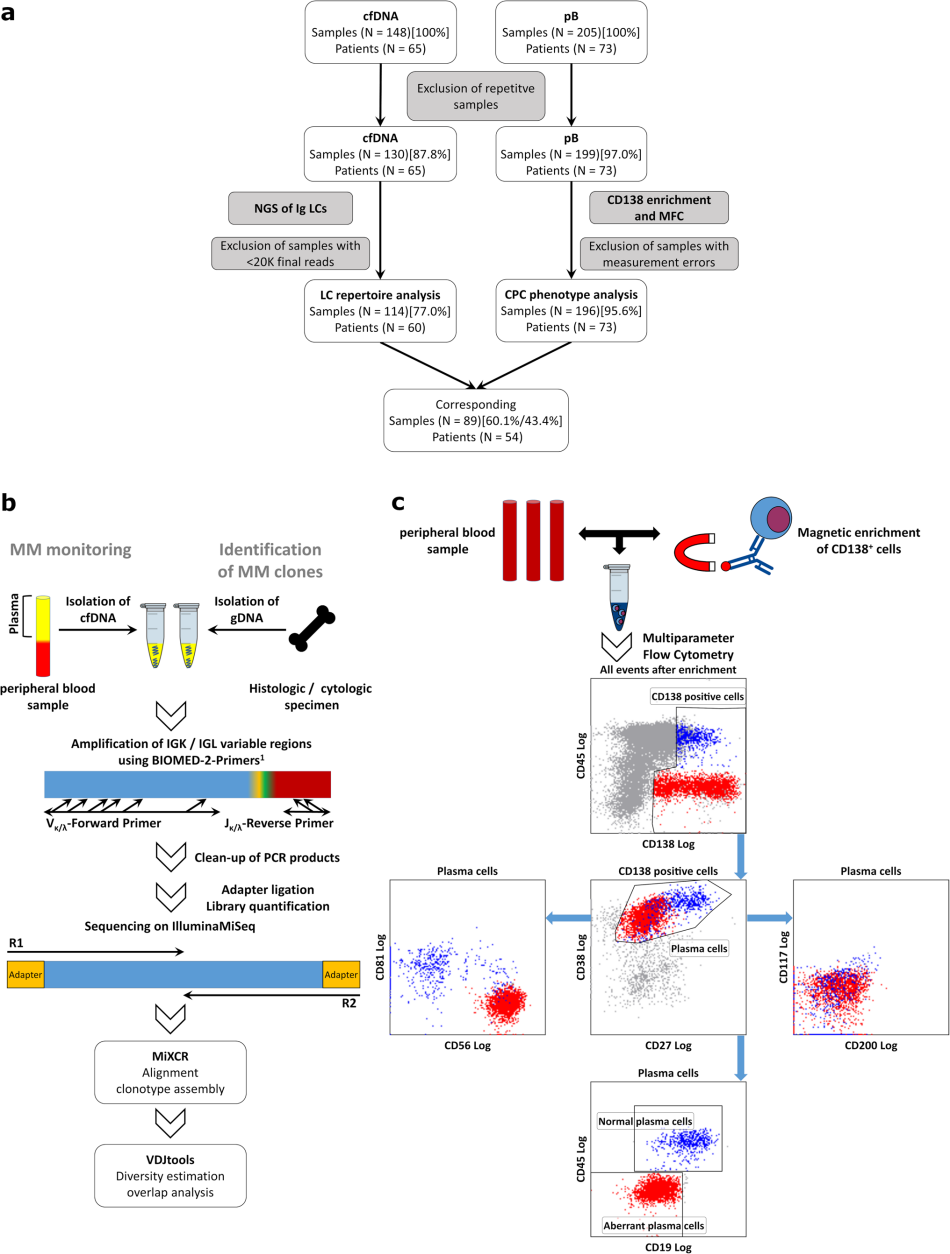
Overview of the final sample sets and analysis strategies. **a** In total, 148 cfDNA samples from 65 patients were collected for NGS of Ig LC repertoires and 205 samples from 73 patients for me-MFC (cf. also Table 1, Table S1). After exclusion of repetitive samples, which had been taken less than 16 days after the previous one, 130 samples (87.8%) were subjected to cfDNA-isolation and NGS and 199 samples (97.0%) to immunomagnetic enrichment of CD138^+^ cells and MFC. Sufficient reads from the involved LC for bioinformatics analysis were obtained for 114 cfDNA samples (77.0%). CPC phenotypes were analyzed for 196 me-MFC samples (95.6%) that could be processed without measurement errors. A subset of 89 corresponding samples (60.1% [NGS] / 43.3% [me-MFC]) was analyzed by both NGS and me-MFC. **b** NGS- and LC repertoire analysis workflows. Libraries were generated by amplification of IGK and IGL variable (VJ) regions using the BIOMED-2 primer sets and sequenced on an Illumina MiSeq instrument with a readlength of 2 × 250 bp. Sequence alignment and clonotype assembly were performed using MiXCR. VDJtools was used for LC repertoire analysis. For confident identification of MM clones, which was not possible based on cfDNA samples alone (cf. main text and supplementary information), histologic or cytologic specimens (bone marrow samples or pleural effusions) were analyzed. **c** Gating strategy for me-MFC analysis: Plasma cells were identified and gated based on expression of CD138, CD38, and CD27. Normal and aberrant plasma cells were discriminated by different expression patterns of CD19, CD45, CD81, CD117, CD56, and CD200. cfDNA, circulating cell-free DNA; me-MFC, magnetic enrichment (of CD138-positive cells) followed by multiparameter flow cytometry; NGS, next generation sequencing; LC, immunoglobulin light chain; CPC, circulating plasma cells

We recruited 80 patients with MM into this study for the analysis of cfDNA by NGS and/or CPCs by me-MFC (Table S1). Initially, we collected 148 cfDNA samples and 205 whole blood samples. After exclusion of repetitive samples that had been taken within less than 16 days from the same patient and samples that yielded insufficient data or measurement errors, 114 samples were available for the analysis of LC repertoires and 196 samples for the analysis of plasma cell immunophenotypes (Fig. 1a). Most samples (86.8% for NGS, 89.3% for me-MFC) were from patients with multiple myeloma, but our series also included samples from patients with oligo-/non-secretory myeloma, plasma cell leukemia, or amyloidosis (Table 1). Moreover, our sample set covered a comprehensive spectrum of response groups according to IMWG criteria, thus allowing us to evaluate NGS of LC repertoires in cfDNA and quantification CPCs in the pB for the estimation of myeloma burden in both serologically detectable and minimal residual disease.

**Table 1:** Overview of the final sample sets analyzed by NGS and/or me-MFC (cf. also Fig. 1, Table S1)

	NGS	me-MFC
**Number of samples analyzed**	114	196
*Type of Myeloma*		
Multiple myeloma (IgG, IgA, LC)	99	175
Smoldering myeloma	1	2
Oligo-/non-secretory myeloma	10	8
Plasma cell leukemia	4	9
Amyloidosis	0	2
*Involved light chain kappa*	*77*	*130*
IgA κ	6	9
IgG κ	42	62
LC κ	28	52
*Involved light chain lambda*	*37*	*66*
IgA λ	17	23
IgG λ	16	24
LC λ	4	17
*Response group*		
ID/RD	18	24
PD	19	19
SD	6	9
PR	15	22
*PR(b)*	*6*	*6*
*PR(a)*	*9*	*16*
VGPR	22	41
CR	34	81

Abbreviations: *NGS*, next generation sequencing; *me-MFC*, magnetic enrichment (of CD138-positive cells) followed by multiparameter flow cytometry; *LC*, immunoglobulin light chain; *ID*, initial diagnosis; *RD*, relapsed disease; *PD*, progressive disease; *SD*, stable disease; *PR(b)*, partial remission before autologous stem cell transplantation; *PR(a)*, partial remission after autologous stem cell transplantation or after > 2 years of therapy; *VGPR*, very good partial remission; *CR*, complete remission

### MM-specific LC rearrangements in cfDNA samples

**Fig. 2:**
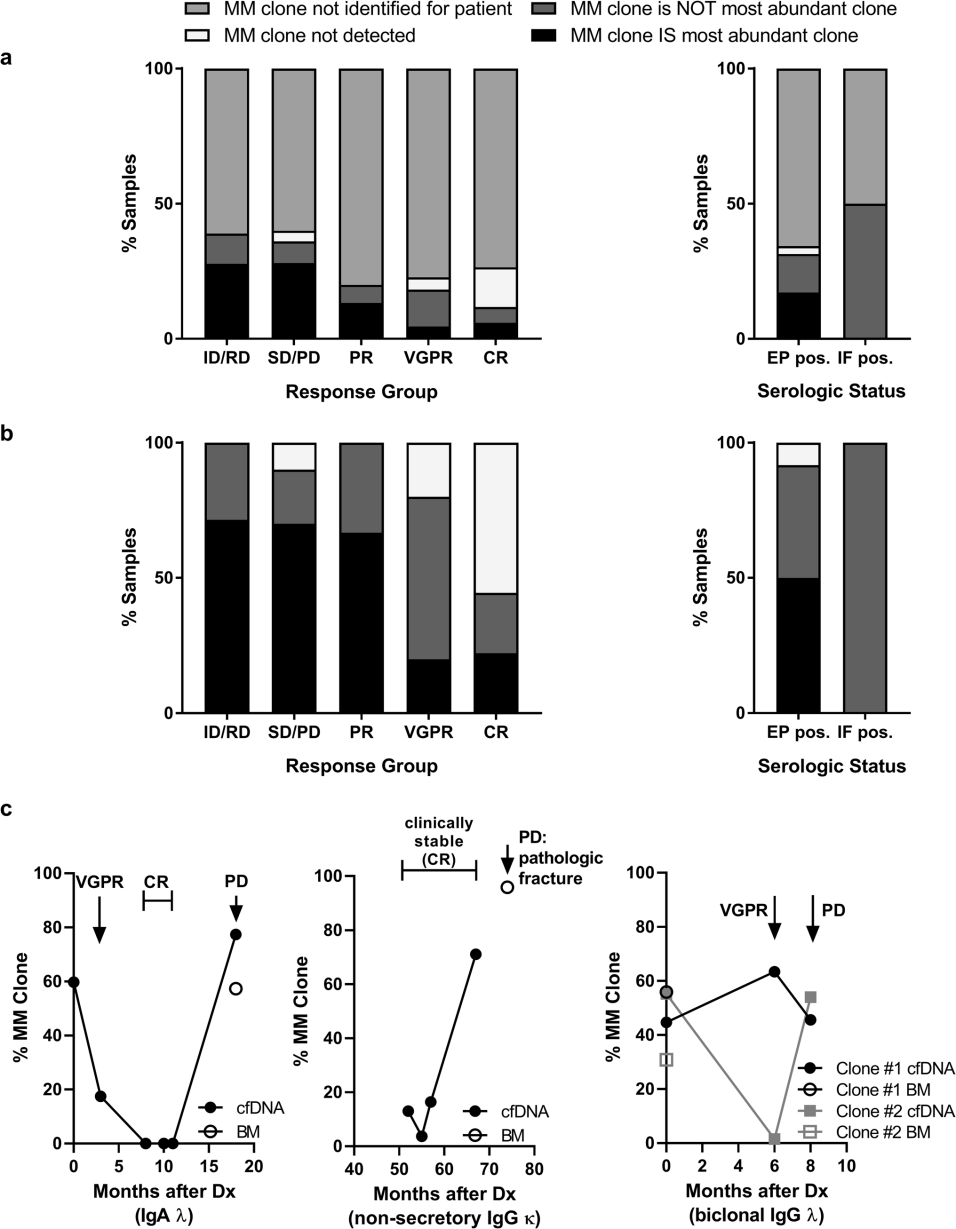
MM-specific LC rearrangements in cfDNA. **a** LC repertoires in cfDNA samples were analyzed using MiXCR. Overview of NGS-results in the complete set of 114 samples according to response group (left panel) and serologic status (right panel). The legend above describing four different result categories also applies to panel (**b**). Sample numbers were *n* = 18 for ID/RD, *n* = 25 for SD/PD, *n* = 15 for PR, *n* = 22 for VGPR, *n* = 34 for CR, *n* = 35 for EP pos., and *n* = 4 for IF pos. **b** Overview of NGS results in the subset of samples from patients, for whom MM clones could be identified according to response group (left panel) and serologic status (right panel). Sample numbers were *n* = 7 for ID/RD, *n* = 10 for SD/PD, *n* = 3 for PR, *n* = 5 for VGPR, *n* = 9 for CR, *n* = 12 for EP pos., and *n*= 2 for IF pos. The fraction of samples with detectable MM clones decreases with deeper remission. **c** Frequencies of putative MM clones over time in patients with different myeloma subtypes. Left panel: MM IgA lambda, middle panel: non-secretory myeloma IgG kappa, right panel: biclonal MM IgG lambda. LC, immunoglobulin light chain; cfDNA, circulating cell-free DNA; NGS, next generation sequencing; ID, initial diagnosis; RD, relapsed disease; PD, progressive disease; SD, stable disease; PR, partial remission; VGPR, very good partial remission; CR, complete remission; EP pos., M-protein detectable on serum electrophoresis; IF pos., positive serum immunofixation and no M protein detectable by serum electrophoresis; MM, multiple myeloma; BM, bone marrow

The NGS assay we used here for the detection of MM in cfDNA (Fig. 1b) focuses on Ig LC rearrangements in order to cover light chain-only MM in addition to MM with rearranged heavy chains. cfDNA was extracted in sufficient amounts for NGS analysis from all samples (*n* = 130, Fig. 1) with no significant differences in yield between response groups (Fig. S1a). For confident analysis of LC spectra, we aimed that repertoires incorporated at least 20,000 reads [33] that could be aligned to the involved LC and assembled into clonotypes by the MiXCR software. A total of 114 samples were sequenced successfully. We observed no significant differences in the diversity of LC repertoires and frequencies of the most abundant clones between response groups (Fig. S1b). This finding clearly suggested that overrepresentation of a clonotype and a reduced or enlarged LC spectrum were not suitable indicators of disease state. Rather, monitoring MM using our genomic LC assay requires exact knowledge on the MM-specific clonotype. The definition of MM-clones is described in detail in the supplementary methods. We identified trackable MM clones for 16 patients (26.7%). MM clones were detected in 23.7% of samples, including 38.9% of ID/RD and 11.8% of CR samples in the total set of 114 samples. The most abundant clonotype corresponded to the MM clone in 27.8% of ID/RD samples, but only in 5.9% of CR samples (Fig. 2a). Considering only samples from patients for whom MM clones were identified (34/114; 29.8%), a MM clone was the most abundant clonotype in 71.4% and 22.2% of ID/RD or, respectively, CR samples. All CR samples in which MM clones were detectable were from a patient with non-secretory MM. No MM clones were found in 55.6% of CR samples (Fig. 2b). Results from serologic studies performed at the time of cfDNA sampling were available only for a subset of samples from patients with MM clones, including 16 serum electrophoresis (EP) samples (4 negative, 12 positive) and 15 immunofixation (IF) samples (2 negative, 13 positive, 2 IF pos./EP neg.). MM clones were detected in 91.7% of the EP positive subset and in 100% of the IF pos./EP neg. samples (Fig. 2b). Although these sample numbers were too small for reliable statistical testing, our findings strongly indicate that NGS of LC repertoires allows for the detection of both serologically measurable and unmeasurable disease if the MM-specific clone is known (Fig. 2c).

### Circulating plasma cells in myeloma patients

**Fig. 3:**
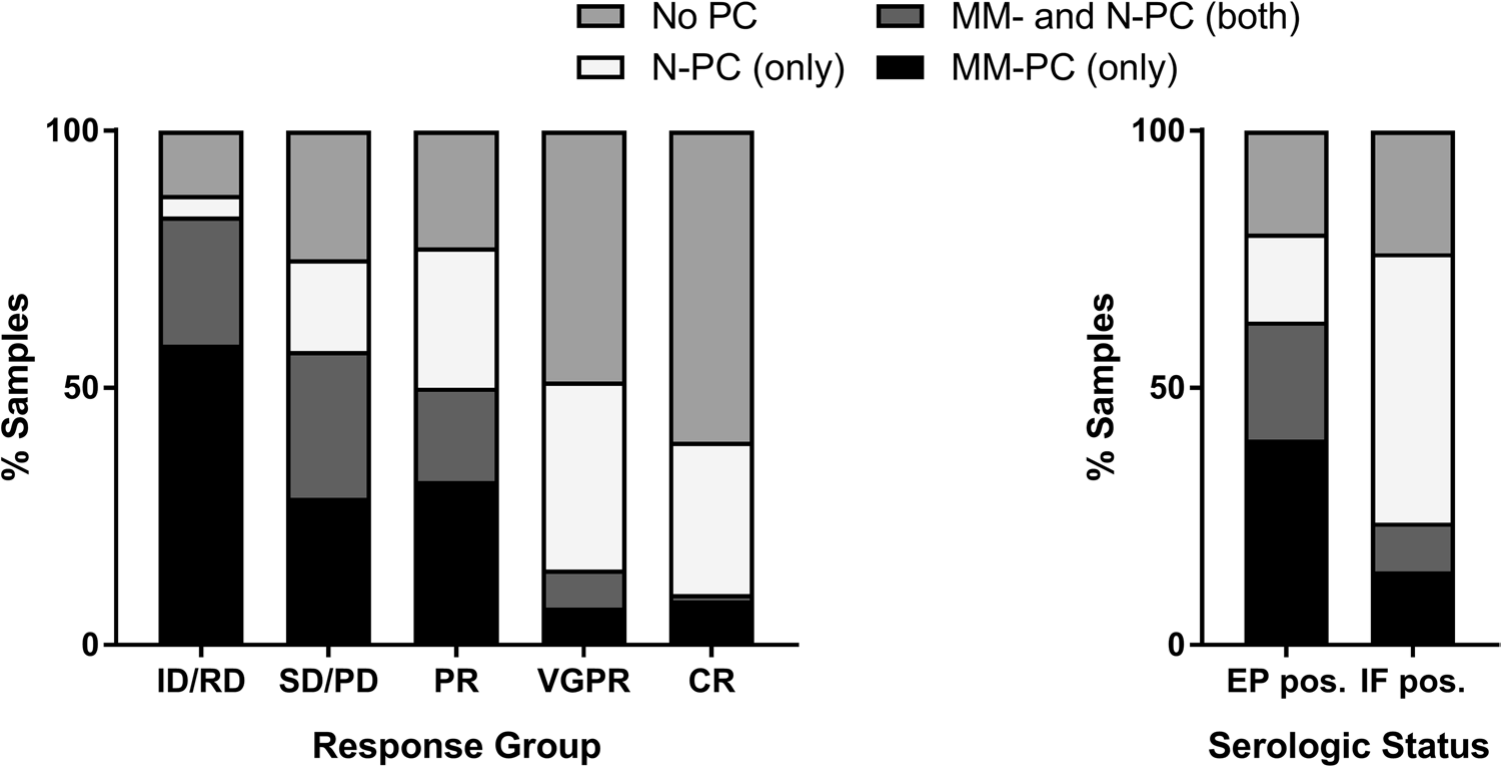
Circulating plasma cells related to MM tumor burden. CPCs were enriched from peripheral blood samples using anti-CD138 magnetic beads and plasma cell phenotypes were analyzed by MFC. Categorical results describing the presence of CPCs were compared according to response group (left panel) or, respectively, to serologic status (right panel). Sample numbers were *n* = 24 for ID/RD, *n* = 28 for SD/PD, *n* = 22 for PR, *n* = 41 for VGPR, *n* = 81 for CR, *n* = 70 for EP pos., and *n* = 21 for IF pos. The fraction of samples with detectable MM-PC decreases with deeper remission. CPCs, circulating plasma cells; MFC, multiparameter flow cytometry; ID, initial diagnosis; RD, relapsed disease; PD, progressive disease; SD, stable disease; PR, partial remission; VGPR, very good partial remission; CR, complete remission; EP pos., M-protein detectable on serum electrophoresis; IF pos., positive serum immunofixation and no M protein detectable by serum electrophoresis; No PC, no plasma cells; N-PC, normal plasma cells; MM-PC, multiple myeloma plasma cells

To examine if MM can be detected based on direct analysis of enriched CPCs, we analyzed the immunophenotypes of CD138^+^ cells separated from the pB via magnetic bead selection by MFC in 196 samples (Fig. 1c). MM-PCs were present in 83.3% of ID/RD samples and decreased with myeloma response to 9.9% of CR samples. On the other hand, 4.2% of ID/RD samples and 29.6% of CR samples exhibited only normal plasma cells. In 12.5% of ID/RD and 60.5% of CR samples, no relevant numbers of CPCs were detected (Fig. 3). In samples taken at time points when EP (and IF, *n* = 70) or IF only (*n* = 21) studies had been positive, MM-PCs were detected in 62.9% and 23.8% of cases, whereas 20.0% and 23.8% of samples did not include CPCs. CR samples positive for MM-PCs were from patients with different types of plasma cell diseases including heavy chain-secreting, light chain-only and non-secretory myeloma. These observations strongly underline the potential of me-MFC to detect residual myeloma cells not only in patients with high disease burden, but also in cases with no evidence of disease as determined by established response parameters.

### LC spectra and plasma cell phenotypes in different types of clinical specimens

**Fig. 4:**
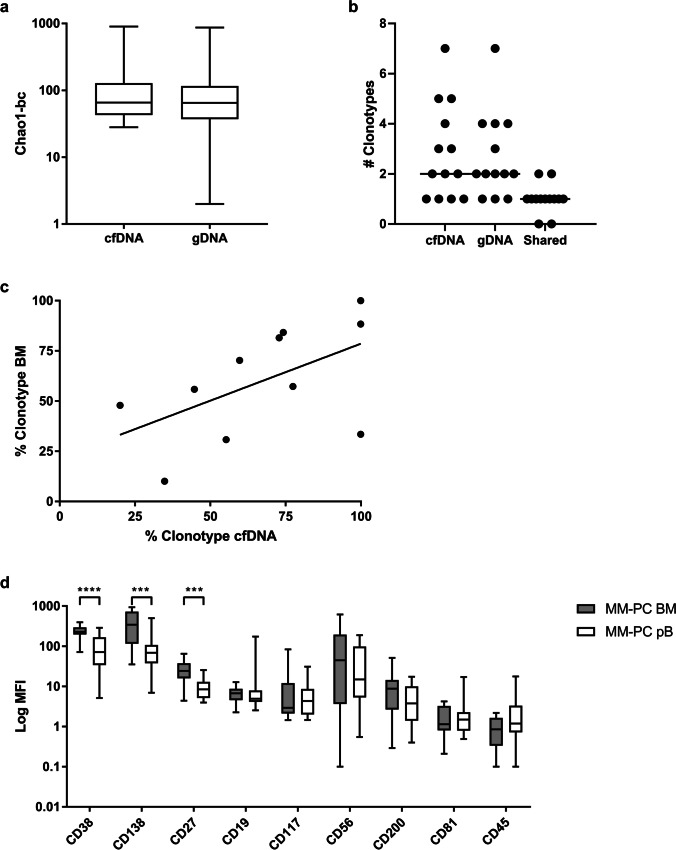
MM Clonotypes and plasma cell immunophenotypes in different sample sources. **a**-**c** Repertoires of the involved LC in MM cfDNA from pB or gDNA samples (obtained from BM biopsies, BM aspirates, or pleural effusions) were analyzed using MiXCR and VDJtools. **a** Diversity of LC spectra as estimated by Chao1-index in samples taken at initial diagnosis. Sample numbers were *n* = 18 for cfDNA and *n* = 21 for gDNA. Diversities were not significantly different by Mann–Whitney test. **b** Overlap analysis of clonotypes accounting > 5% of the LC spectra were compared between gDNA and corresponding cfDNA samples. *n* = 13; 9 sample pairs (69.2%) displayed only one shared clonotype. **c** Correlation of clonotype frequencies of MM clones in corresponding cfDNA and gDNA samples from patients for whom MM clones could be identified. *n* = 11. There was a significant correlation of MM clonotype frequencies in samples from different sources; Spearman *r* = 0.6147, *p* = 0.0498. **d** Comparison of plasma cell phenotypes from pB (CD138 selected) and paired BM (not selected) samples. *n* = 17. MFI for each surface marker was compared statistically using paired *t*-tests and the Holm-Sidak method to adjust *p*-values. Only significant differences are indicated. ***p < 0.001, ****p < 0.0001. MFI was significantly higher for CD38, CD138, and CD27 in BM samples than in pB samples of the same patients. LC, immunoglobulin light chain; MM, multiple myeloma; cfDNA, circulating cell-free DNA; pB, peripheral blood; BM, bone marrow; gDNA, genomic DNA; MFI, Mean fluorescence intensity; MM-PC, multiple myeloma plasma cells

Our findings stated above provide a sound proof-of-principle that NGS of LC repertoires in cfDNA and me-MFC of CPCs allow for the detection of MRD in MM, but we also note that both methods are not universally applicable for the quantification of disease burden in MM. Specifically, we did not identify strongly overrepresented clonotypes comprising > 33.3% of the LC spectrum in 7 of 18 (38.9%) cfDNA samples or, respectively, could not detect aberrant CPCs by magnetic bead selection in 3/24 (12.5%) pB samples taken at ID/RD. We therefore compared LC spectra and plasma cell phenotypes in peripheral blood to histologic/cytologic MM samples in more detail. Diversity of LC spectra was not significantly different between diagnostic cfDNA and BM samples (Fig. 4a). Moreover, we found a significant correlation of the frequencies of MM clones in cfDNA and BM biopsies (Spearman *r* = 0.6147, *p* = 0.0498), although there were generally only minor overlaps of LC spectra in cfDNA and other types of MM samples comprising a median of only one clonotype accounting for > 5% of the LC spectrum in both specimens (Fig. 4b, c). Comparing CPC immunophenotypes, we observed a significantly higher expression of CD38 (*p* < 0.001), CD138 (*p* < 0.01), and CD27 (*p* < 0.01) in BM plasma cells compared to CPCs.

These observations indicate that the representation of MM in the peripheral blood by cfDNA is somehow biased by distinct molecular properties that make the disease inaccessible to detection by NGS of LC repertoires at least at some time points and/or in a subset of patients. This goes along with a divergent immunophenotype of CPCs compared to BM plasma cells, which further underlines biological differences in MM-PCs from various compartments.

### Concordance of NGS and me-MFC results to estimate MM burden

**Fig. 5:**
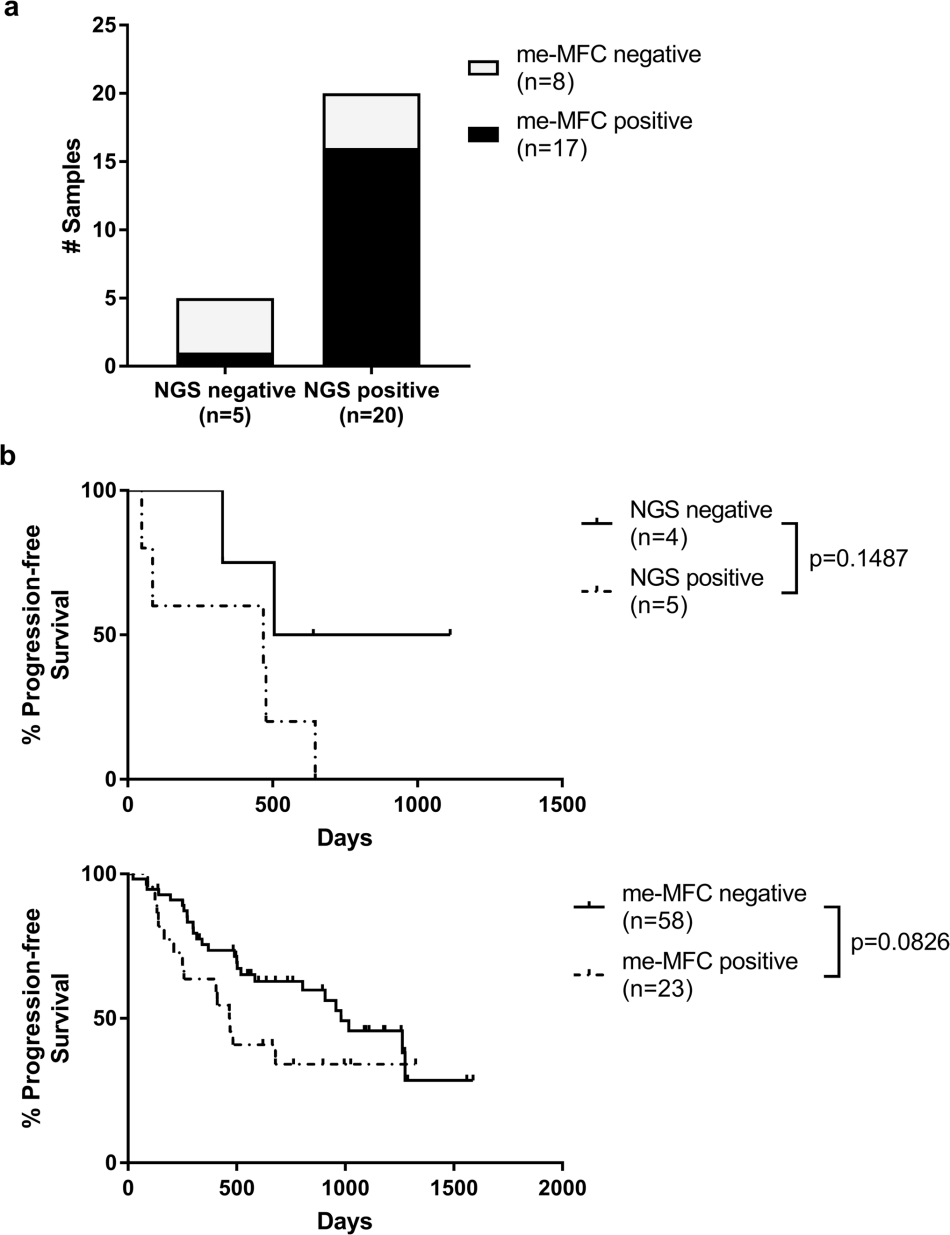
Concordance and clinical implications of LC repertoire analysis in cfDNA and me-MFC for monitoring MM. **a** 25 corresponding cfDNA and whole blood samples obtained from the same patients (with MM clones) at the same time points were analyzed for the concordance of NGS and me-MFC results. All samples in which MM clones were detected irrespective of frequency were classified as “NGS positive,” and all samples without detectable MM clones as “NGS negative.” me-MFC samples containing MM-PC only or both MM-PC and N-PC were classified as “MFC-positive,” and samples containing only N-PC or no plasma cells as “MFC negative.” **b** Progression-free survival of MM patients according to results of NGS (upper panel) and me-MFC (lower panel) analyses. Days are counted from the day of sampling. If more than one sample was available for a patient before progression and me-MFC/NGS results were identical for all of these samples, only the earliest sample was included in the analysis. NGS samples were from patients with MM clones only. NGS and me-MFC samples were categorized as positive/negative as in (**a**). Survival times were not significantly different by Log-rank test. cfDNA, circulating cell-free DNA; MM, multiple myeloma; NGS, next generation sequencing; me-MFC, magnetic enrichment (of CD138-positive cells) followed by multiparameter flow cytometry; MM-PC, multiple myeloma plasma cells; N-PC, normal plasma cells

Given that estimation of MM disease burden by the two experimental approaches developed in this work obviously is — at least in part — dependent on biological properties of the disease itself, we analyzed the agreement of NGS and me-MFC results. Our sample series included 89 corresponding samples taken at the same time point from the same patients that were subjected to both NGS and me-MFC. However, since exact knowledge of the MM clone is essential for quantification of MM disease burden by NGS of LC repertoires, we only compared NGS and me-MFC results in 25 samples from patients with MM clones. For this purpose, we classified all samples in which MM clones were detected (irrespective of frequency) as “NGS positive,” and all samples, in which we did not detect MM clones as “NGS negative.” Regarding me-MFC, samples containing MM-PC only or both MM-PC and normal plasma cells (N-PC) were considered “MFC-positive,” while samples in which we detected only N-PC or no PCs were categorized as “MFC negative.” NGS and me-MFC results were concordant for 20 samples (80%), corresponding to a Cohen’s Kappa value of 0.490 (Fig. 5a). Thus, there was only a moderate agreement between NGS-and me-MFC, further underlining that the two methods provide non-redundant information on the disease state.

### Potential prognostic implications of LC repertoire analysis in cfDNA and me-MFC for monitoring MM

Assuming that quantification of clonotypic rearrangements in cfDNA and aberrant plasma cells in the circulation are complimentary rather than equivalent measures of MM burden not only with respect to each other but also to established myeloma parameters, we also explored potential prognostic implications of positive, or respective negative results from our two experimental approaches to monitoring MM. Because our cohort was highly heterogeneous concerning disease/remission status and prior therapies, we chose to analyze progression-free survival. For these analyses, nine cfDNA samples from patients with MM clones and 81 me-MFC samples were available. Progression-free survival was longer when there was no evidence for MM by NGS or me-MFC, yet PFS-gains were not significant (Fig. 5b). These observations strongly point out that minimally-invasive estimates of MM burden may be valuable for risk-reevaluations in the course of the disease, although our findings need clarifications in larger patient cohorts that also allow stratification by remission, particularly in the CR subgroup, i.e., in the setting of MRD.

## Discussion

In this work, we designed and initially validated two analytical approaches to investigate disease burden in MM in pB samples. Firstly, we modified the well-established PCR approach for B-cell clonality analyses developed by the BIOMED-2 working group for NGS of cfDNA. Different from a standardized Ig sequencing protocol published only recently by this working group [34] and from the original protocol, we included both IGK and IGL loci and combined all primers for a specific LC in a single tube for target amplification. Previous studies examining the course of MM using liquid biopsies investigated either heavy chains only or both heavy and light chains [18–22, 35, 36]. Secondly, we adapted a semiautomatic magnetic bead-based cell selection procedure developed in our laboratory [37] for enrichment of CD138^+^ cells from pB prior to MFC analysis. Thus, the work presented here — to the best of our knowledge — includes the first parallel evaluation of two technically different diagnostic procedures for minimally invasive monitoring of MM.

The major prerequisite for clinical applicability of a liquid biopsy-based assay is the presence of the target in the pB. Here, we showed that cfDNA can be isolated in sufficient amounts for sequencing studies at all stages of disease. Given that mutational landscapes of MM are heterogeneous even in one individual’s disease [38–41], clonal rearrangements represent the preferred target for sequencing analyses as these have been found to be stable over time and closely related in all MM subclones [36]. In our work, overlap analysis of clone spectra in cfDNA and FFPE/gDNA samples was extremely helpful to identify MM clones, particularly since LC repertoires exhibited several high frequency clones at any stage of disease. While this observation may be explained by insufficient clearance of the malignant clone in partial responders, mono- and oligo- instead of polyclonality in CR samples might mirror ongoing immune reconstitution after intensive and effective treatment, which can even lead to false positive and/or atypical immunofixation results [26, 42]. Yet, sequencing of BM biopsies may also fail to detect MM clones due to an inhomogeneous infiltration pattern [43] or technical difficulties arising from low-quality DNA [44] or impaired primer annealing [44–46]. Therefore, identification of high evidence myeloma clones should include either sorted CD138-positve cells [18, 19, 35, 36] or several samples from different sources including at least one specimen with a high content of aberrant plasma cells. Although the detection of residual MM in our work may have been hampered due to lack of knowledge of the specific MM clone resulting from limited availability of samples from different sources for the majority of patients and presumably from restricting our assay to light chains only [20, 34], our findings are also in line with a previous report stating that not all MMs release DNA into the plasma [20]. Similarly, circulating MM-PCs are not found in all cases of MM even at high disease burden as these represent only a subset of MM plasma cells that has been described to be phenotypically, genetically, and functionally distinct from BM plasma cells [47]. Despite evasion of the BM into pB may be a biological property of distinct MM subclones only and therefore not been observed universally in MM patients, we detected MM-PCs in 19 of 22 (86.3%) of ID samples by me-MFC. Thus, our cell-based approach achieved higher patient coverage than the NGS assay. The me-MFC assay, which also does not require a second sample from a different source for confident identification of the target to track, may therefore be more suitable for monitoring MM in the pB in a clinical routine setting than the detection of clonal Ig rearrangements. Of note, in the study described here, we have not explicitly evaluated the sensitivity of me-MFC compared to bulk approaches so that we cannot ultimately decide whether materials and time efforts used for the magnetic preenrichment step actually productively improve the MFC assay for MM detection. Beyond that, we have not studied whether the assay, when used during follow-up, needs adaption of the antibody panel to avoid loss of aberrant CPCs with downregulation of CD38 after CD38-antibody treatment [48]. On the other hand, both NGS of LC repertoires in cfDNA and me-MFC detected evidence of residual disease in samples taken at time points of good remission (VGPR and CR), thus in principle underlining the usability of either approach for MRD monitoring. Interestingly, all four CR samples that were MRD-positive by NGS and five of eight CR samples with detectable MM-PCs by me-MFC were from patients with non-secretory or oligo-secretory myeloma, i.e., subtypes of myeloma that are difficult to track by standard clinical and laboratory investigations. We observed a moderate agreement of NGS and me-MFC results, which is in line with the current literature as perfect consistency of different MRD assays involving different analytical approaches and/or different sample materials such as NGS vs. MFC or pB vs BM is not expected based on previous reports [18, 33, 49]. Finally, our data provide a first hint that the presence of clonotypic LC rearrangements in cfDNA and/or aberrant plasma cells in the pB are associated with an inferior prognosis. Taken together, our preliminary validation studies of two technical modifications of molecular and cell-based assays for detecting MM in the pB support a future perspective to develop gradual follow-up concepts for MM patients that schedule investigations of BM samples only when there is no evidence of disease in the pB. However, the pilot study presented here clearly is not sufficient to boost the so far only emerging clinical utility of liquid biopsies in MM [25, 50]. Further studies in larger patient cohorts that directly compare NGS- and MFC-approaches are needed to fully establish the preferred method for minimally invasive monitoring of MM.

## Supplementary Information

Below is the link to the electronic supplementary material.

Supplementary file1 (DOCX 295 KB)

## Data Availability

All sequencing and bioinformatical data are available from the authors upon request.

## References

[1] Ghandili S, Weisel KC, Bokemeyer C, Leypoldt LB (2021). Current treatment approaches to newly diagnosed multiple myeloma. Oncol Res Treat.

[2] Marneni N, Chakraborty R (2021). Current approach to managing patients with newly diagnosed high-risk multiple myeloma. Curr Hematol Malig Rep.

[3] Rajkumar SV (2021). Sequencing of myeloma therapy: finding the right path among many standards. Hematol Oncol.

[4] Gengenbach L, Graziani G, Reinhardt H (2021). Choosing the right therapy for patients with relapsed/refractory multiple myeloma (RRMM) in consideration of patient-, disease- and treatment-related factors. Cancers (Basel).

[5] Dimopoulos MA, Jakubowiak AJ, McCarthy PL (2020). Developments in continuous therapy and maintenance treatment approaches for patients with newly diagnosed multiple myeloma. Blood Cancer J.

[6] Tang F, Malek E, Math S (2018). Interference of therapeutic monoclonal antibodies with routine serum protein electrophoresis and immunofixation in patients with myeloma: frequency and duration of detection of daratumumab and elotuzumab. Am J Clin Pathol.

[7] Bravo-Pérez C, Sola M, Teruel-Montoya R, et al (2021) Minimal residual disease in multiple myeloma: something old, something new. Cancers 13 10.3390/cancers13174332 PMC8430644 34503142

[8] Rodriguez-Otero P, Paiva B, San-Miguel JF (2021) Roadmap to cure multiple myeloma. Cancer Treat Rev 100:. 10.1016/j.ctrv.2021.102284 10.1016/j.ctrv.2021.102284 34597912

[9] Haber DA, Velculescu VE (2014) Blood-based analyses of cancer: circulating tumor cells and circulating tumor DNA. Cancer Discov 4:650 LP – 661 10.1158/2159-8290.CD-13-1014 PMC4433544 24801577

[10] Mithraprabhu S, Khong T, Ramachandran M (2017). Circulating tumour DNA analysis demonstrates spatial mutational heterogeneity that coincides with disease relapse in myeloma. Leukemia.

[11] Kis O, Kaedbey R, Chow S (2017). Circulating tumour DNA sequence analysis as an alternative to multiple myeloma bone marrow aspirates. Nat Commun.

[12] Gonsalves WI, Morice WG, Rajkumar V (2014). Quantification of clonal circulating plasma cells in relapsed multiple myeloma. Br J Haematol.

[13] Nowakowski GS, Witzig TE, Dingli D (2005). Circulating plasma cells detected by flow cytometry as a predictor of survival in 302 patients with newly diagnosed multiple myeloma. Blood.

[14] Zhang L, Beasley S, Prigozhina NL (2016). Detection and characterization of circulating tumour cells in multiple myeloma. J Circ biomarkers.

[15] Wang N, Tesfaluul N, Li J (2019). Enrichment of circulating myeloma cells by immunomagnetic beads combined with flow cytometry for monitoring minimal residual disease and relapse in patients with multiple myeloma. Ann Hematol.

[16] Qasaimeh MA, Wu YC, Bose S (2017). Isolation of circulating plasma cells in multiple myeloma using CD138 Antibody-based capture in a microfluidic device. Sci Rep.

[17] Foulk B, Schaffer M, Gross S (2018). Enumeration and characterization of circulating multiple myeloma cells in patients with plasma cell disorders. Br J Haematol.

[18] Mazzotti C, Buisson L, Maheo S (2018). Myeloma MRD by deep sequencing from circulating tumor DNA does not correlate with results obtained in the bone marrow. Blood Adv.

[19] Vij R, Mazumder A, Klinger M (2014). Deep sequencing reveals myeloma cells in peripheral blood in majority of multiple myeloma patients. Clin Lymphoma, Myeloma Leuk.

[20] Oberle A, Brandt A, Voigtlaender M (2017). Monitoring multiple myeloma by next-generation sequencing of V(D)J rearrangements from circulating myeloma cells and cell-free myeloma DNA. Haematologica.

[21] Biancon G, Gimondi S, Vendramin A (2018). Noninvasive molecular monitoring in multiple myeloma patients using cell-free tumor DNA: a pilot study. J Mol Diagnostics.

[22] Korthals M, Sehnke N, Kronenwett R (2013). Molecular monitoring of minimal residual disease in the peripheral blood of patients with multiple myeloma. Biol Blood Marrow Transplant.

[23] Manier S, Park J, Capelletti M (2018). Whole-exome sequencing of cell-free DNA and circulating tumor cells in multiple myeloma. Nat Commun.

[24] Mithraprabhu S, Morley R, Khong T (2019). Monitoring tumour burden and therapeutic response through analysis of circulating tumour DNA and extracellular RNA in multiple myeloma patients. Leukemia.

[25] Mithraprabhu S, Chen M, Savvidou I (2021). Liquid biopsy: an evolving paradigm for the biological characterisation of plasma cell disorders. Leukemia.

[26] Kumar S, Paiva B, Anderson KC (2016). International Myeloma Working Group consensus criteria for response and minimal residual disease assessment in multiple myeloma. Lancet Oncol.

[27] van Dongen JJM, Langerak AW, Bruggemann M, et al Design and standardization of PCR primers and protocols for detection of clonal immunoglobulin and T-cell receptor gene recombinations in suspect lymphoproliferations: report of the BIOMED-2 Concerted Action BMH4-CT98–3936. Leukemia 17:2257–2317 10.1038/sj.leu.2403202 14671650

[28] Bolotin DA, Poslavsky S, Mitrophanov I (2015). MiXCR: software for comprehensive adaptive immunity profiling. Nat Methods.

[29] Shugay M, Bagaev DV, Turchaninova MA (2015). VDJtools: unifying post-analysis of T cell receptor repertoires. PLOS Comput Biol.

[30] Colwell RK, Chao A, Gotelli NJ (2012). Models and estimators linking individual-based and sample-based rarefaction, extrapolation and comparison of assemblages. J Plant Ecol.

[31] Chao A (1984). Nonparametric estimation of the number of classes in a population. Scand J Stat.

[32] Chaudhary N, Wesemann DR (2018). Analyzing immunoglobulin repertoires Front Immunol.

[33] Medina A, Puig N, Flores-Montero J (2020). Comparison of next-generation sequencing (NGS) and next-generation flow (NGF) for minimal residual disease (MRD) assessment in multiple myeloma. Blood Cancer J.

[34] Brüggemann M, Kotrová M, Knecht H (2019). Standardized next-generation sequencing of immunoglobulin and T-cell receptor gene recombinations for MRD marker identification in acute lymphoblastic leukaemia; a EuroClonality-NGS validation study. Leukemia.

[35] Korde N, Mailankody S, Roschewski M (2014). Minimal residual disease (MRD) testing in newly diagnosed multiple myeloma (MM) patients: a prospective head-to-head assessment of cell-based, molecular, and molecular-imaging modalities. Blood.

[36] Rustad EH, Misund K, Bernard E (2019). Stability and uniqueness of clonal immunoglobulin CDR3 sequences for MRD tracking in multiple myeloma. Am J Hematol.

[37] Hoffmann JC, Stabla K, Burchert A (2014). Monitoring of acute myeloid leukemia patients after allogeneic stem cell transplantation employing semi-automated CD34+ donor cell chimerism analysis. Ann Hematol.

[38] Rasche L, Chavan SS, Stephens OW (2017). Spatial genomic heterogeneity in multiple myeloma revealed by multi-region sequencing. Nat Commun.

[39] Paíno T, Paiva B, Sayagués JM (2015). Phenotypic identification of subclones in multiple myeloma with different chemoresistant, cytogenetic and clonogenic potential. Leukemia.

[40] Lohr JG, Stojanov P, Carter SL (2014). Widespread genetic heterogeneity in multiple myeloma: implications for targeted therapy. Cancer Cell.

[41] Mishima Y, Paiva B, Shi J (2017). The mutational landscape of circulating tumor cells in multiple myeloma. Cell Rep.

[42] Mark T, Jayabalan D, Coleman M (2008). Atypical serum immunofixation patterns frequently emerge in immunomodulatory therapy and are associated with a high degree of response in multiple myeloma. Br J Haematol.

[43] Zamagni E, Tacchetti P, Barbato S, Cavo M (2020). Role of imaging in the evaluation of minimal residual disease in multiple myeloma patients. J Clin Med.

[44] Scheijen B, Meijers RWJ, Rijntjes J (2019). Next-generation sequencing of immunoglobulin gene rearrangements for clonality assessment: a technical feasibility study by EuroClonality-NGS. Leukemia.

[45] Ladetto M, Brüggemann M, Monitillo L (2014). Next-generation sequencing and real-time quantitative PCR for minimal residual disease detection in B-cell disorders. Leukemia.

[46] Bai Y, Orfao A, Chim CS (2018). Molecular detection of minimal residual disease in multiple myeloma. Br J Haematol.

[47] Rawstron AC, Owen RG, Davies FE (1997). Circulating plasma cells in multiple myeloma: characterization and correlation with disease stage. Br J Haematol.

[48] Broijl A, de Jong ACM, van Duin M, et al (2021) VS38c and CD38-multiepitope antibodies provide highly comparable minimal residual disease data in patients with multiple myeloma. Am J Clin Pathol aqab163. 10.1093/ajcp/aqab163 10.1093/ajcp/aqab163 PMC8973271 34643211

[49] Sanoja-Flores L, Flores-Montero J, Puig N (2019). Blood monitoring of circulating tumor plasma cells by next generation flow in multiple myeloma after therapy. Blood.

[50] Yee AJ, Raje N (2021). Minimal residual disease in multiple myeloma: why, when, where. Hematology.

