# Supplementary material for: Monitoring multiple myeloma in the peripheral blood based on cell-free DNA and circulating plasma cells
Source: Ann Hematol. 2022 Feb 1;101(4):811–24. doi: 10.1007/s00277-022-04771-5 (PMC8913458; doi:10.1007/s00277-022-04771-5)
Supplement: Supplementary file 1 — Supplementary file1 (DOCX 295 KB) [file 277_2022_4771_MOESM1_ESM.docx]

**Supplementary Information**

**Monitoring Multiple Myeloma in the peripheral blood based on cell-free DNA and circulating plasma cells**

Elisabeth K.M. Mack, Sören Hartmann, Petra Ross, Ellen Wollmer, Christoph Mann, Andreas Neubauer, Cornelia Brendel, and Jörg Hoffmann

**Contents**

Supplementary Methods

Supplementary Figure S1

Supplementary Table S1

**Supplementary Methods**

*Histologic/cytologic specimens for NGS*

Genomic DNA samples were from bone marrow (BM) biopsies (n=35), BM aspirates (n=2) or pleural effusions (n=1). Sufficient data (>20.000 reads for the involved LC) for confident repertoire analysis were obtained for 34 samples. Remission states at the time of sample collection were ID (n=21), PD (n=9), SD (n=1), PR (n=2) and CR (n=1; non-secretory myeloma).

*Identification of potential MM clones*

Our analyses of LC spectra in cfDNA of MM patients revealed no significant differences in diversity or frequencies of the most abundant clonotypes between IMWG response groups (cf. main text and Figure S1b), indicating that the most abundant clone in each sample not necessarily corresponded to the malignant MM clone. However, we noted a clear separation of PR samples according to the time in the course of the disease when the PR was achieved, as early PR samples obtained before autologous stem cell transplantation (PR(b)) were the only subset that significantly differed from ID/RD samples in diversity and frequencies of the most abundant clonotype (Figure S1c). Therefore, in order to identify specific LC rearrangements that very likely originated from the malignant clone (referred to as potential MM clones in the following), we initially focused on rearrangements that comprised >33.3% of the involved LC repertoires in ID/RD, SD/PD and PR(b) samples. In total, 37 of such highly overrepresented clonotypes were present in 32 of 39 samples from 32 patients, but only 15 patients had more than one cfDNA sample available and only seven clonotypes could be detected repeatedly in different samples from the same patient. Thus, potential MM clones could not be deduced from cfDNA samples alone for most patients so that we additionally examined Ig LC repertoires in 34 histological or cytological specimens. By careful cross-comparison of LC spectra in cfDNA and gDNA samples, we developed the following criteria to determine trackable potential myeloma clones: 1) The clonotype must be detectable in at least two samples – one of which at a time of no/poor response (ID/RD, SD/PD and PR(b)); 2) The clonotype must be overrepresented – i.e. represent >33.3% of the LC repertoire in at least one or >20% in at least two no/poor response samples of a given patient; 3) *Biclonality*: Samples were considered biclonal if they showed two different >10% rearrangements in at least two samples – one of which at no/poor response. Applying these criteria, we identified MM clones in 16 patients, with 13 patients exhibiting one, and three patients two MM clones.

**Supplementary Table**

**Table S1: Overview of patients from whom samples were obtained for this study.**

(Table accompanies Table 1 and Figure 1)

|  | **NGS** | **me-MFC** |
| --- | --- | --- |
| **Number of patients** | 65 | 73 |
| Age at diagnosis [years], mean (range) | 60 (36-79) | 60 (36-79) |
| *Gender* |  |  |
| Female | 29 | 33 |
| Male | 36 | 40 |
| *Type of Myeloma* |  |  |
| Multiple Myeloma (IgG, IgA, LC) | 56 | 64 |
| Smoldering Myeloma | 3 | 2 |
| Oligo-/Non-secretory Myeloma | 4 | 4 |
| Plasma Cell Leukemia | 2 | 2 |
| Amyloidosis | 0 | 1 |
| *Involved light chain kappa* | *41* | *49* |
| IgA κ | 5 | 6 |
| IgG κ | 24 | 28 |
| LC κ | 12 | 14 |
| *Involved light chain lambda* | *24* | *24* |
| IgA λ | 9 | 8 |
| IgG λ | 12 | 11 |
| LC λ | 3 | 4 |

Abbreviations: NGS: next generation sequencing. me-MFC: magnetic enrichment (of CD138-positive cells) followed by multiparameter flow cytometry

**Supplementary Figure**

**
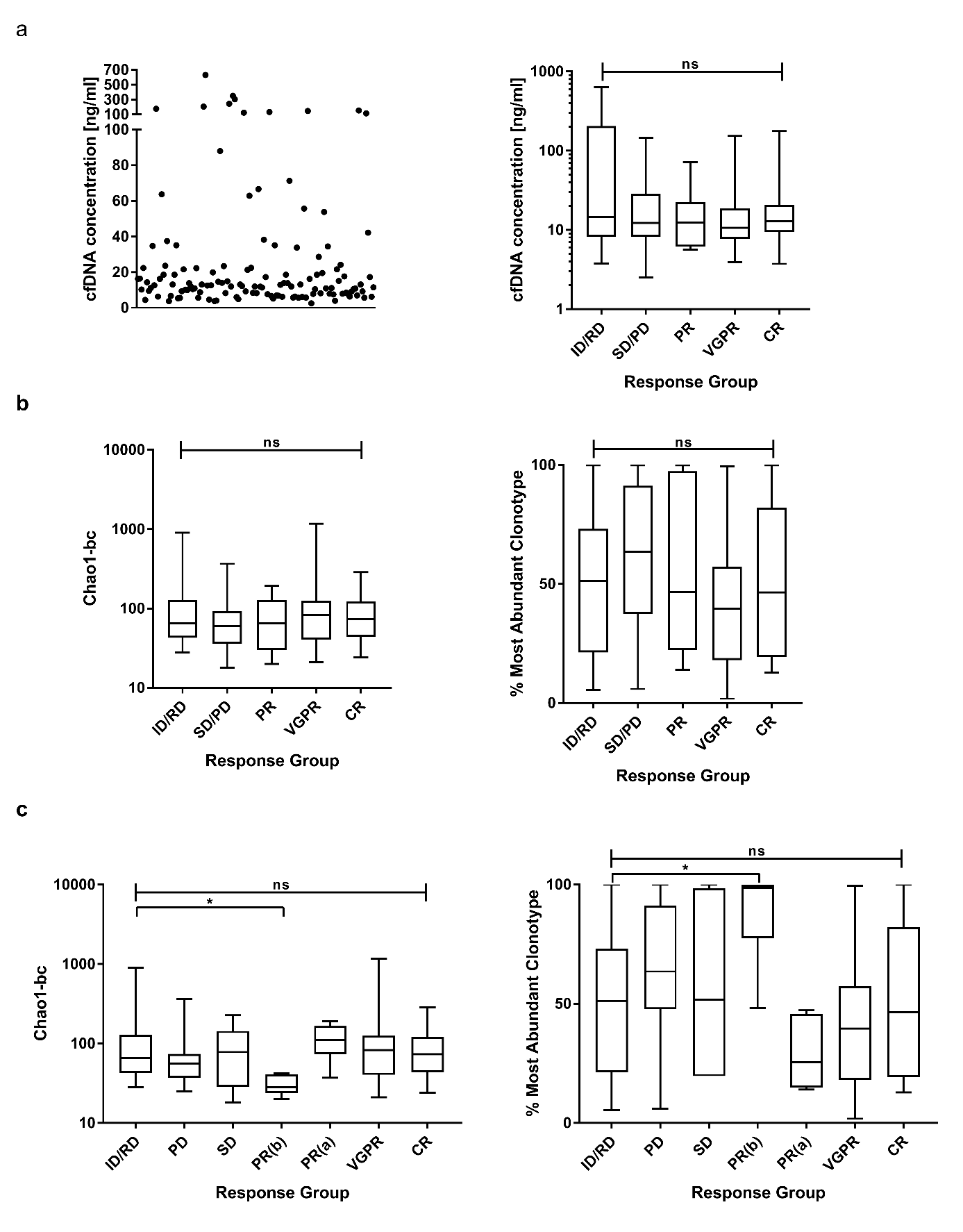
**

**Fig. S1 cfDNA concentrations and diversity of LC clone spectra in MM patients**

(a) cfDNA was isolated from 130 peripheral blood samples (cf. Fig. 1) and patient plasma concentrations were calculated from the concentrations of the eluates assuming a plasma volume of 5 ml. Sample numbers were n=19 for ID/RD, n=31 for SD/PD, n=18 for PR, n=26 for VGPR, n=36 for CR. (b, c) LC repertoires for n=114 samples, for which >20,000 reads were obtained by NGS, were analyzed using MiXCR and VDJtools. Clonotype diversity (Chao1-bc index, left panels) and frequencies of the most abundant clonotypes (right panels) are indicated. PR samples separated in two groups depending on when the PR was achieved (PR(b) and PR(a), see abbreviation list below). Sample numbers were n=18 for ID/RD, n=19 for PD, n=6 for SD, n=6 for PR(b), n=9 for PR(a), n=22 for VGPR, n=34 for CR. Box and whiskers-plots represent medians with 25th to 75th percentile and the range of values. Kruskal-Wallis-test with Dunn’s multiple comparisons test between each response group and the ID/RD group was used for statistical comparisons. For the post tests, only significant results are indicated. cfDNA: circulating cell-free DNA. ID: initial diagnosis. RD: relapsed disease. PD: progressive disease. SD: stable disease. PR: partial remission. VGPR: very good partial remission. CR: complete remission. LC: immunoglobulin light chain. NGS: next generation sequencing. . PR(a): partial remission after autologous stem cell transplantation or after >2 years of therapy.
